# Supplementary material for: Multiepitope fusion protein-based ELISA for enhanced brucellosis serodiagnosis
Source: PLoS Negl Trop Dis. 2025 Dec 3;19(12):e0013804. doi: 10.1371/journal.pntd.0013804 (PMC12685213; doi:10.1371/journal.pntd.0013804)
Supplement: S3 File — (DOCX) [file pntd.0013804.s003.docx]

**S3 File. Original images for Fig 2.**

**
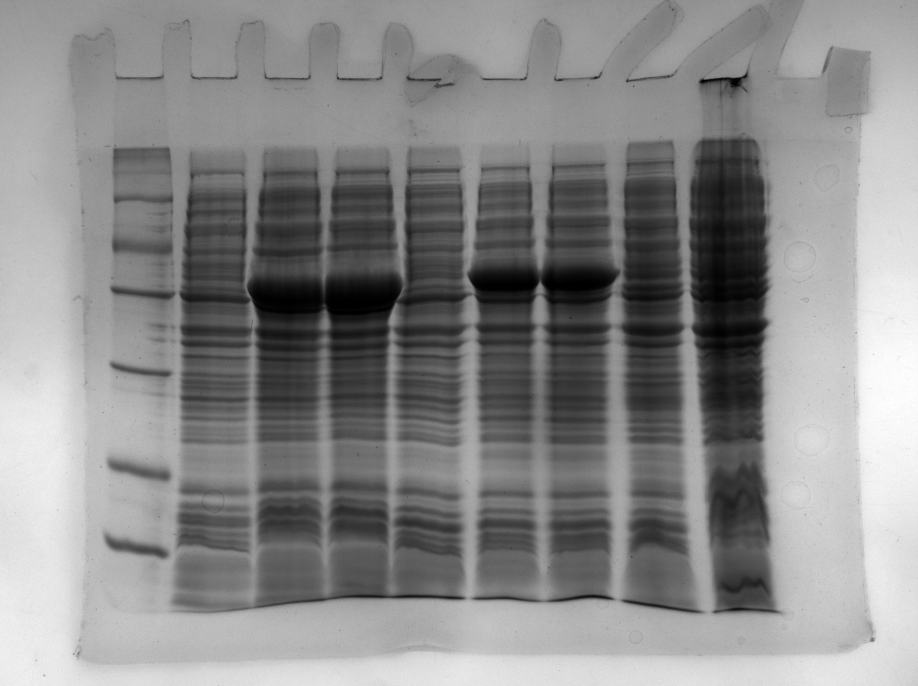
**

**M 1 2 3**

**Fig A. Original image for protein expression results in small amounts.**

M, Marker; Lane 1, Uninduced control (BL21); Lane 2-3, IPTG induction (BL21). The rest are irrelevant samples

**
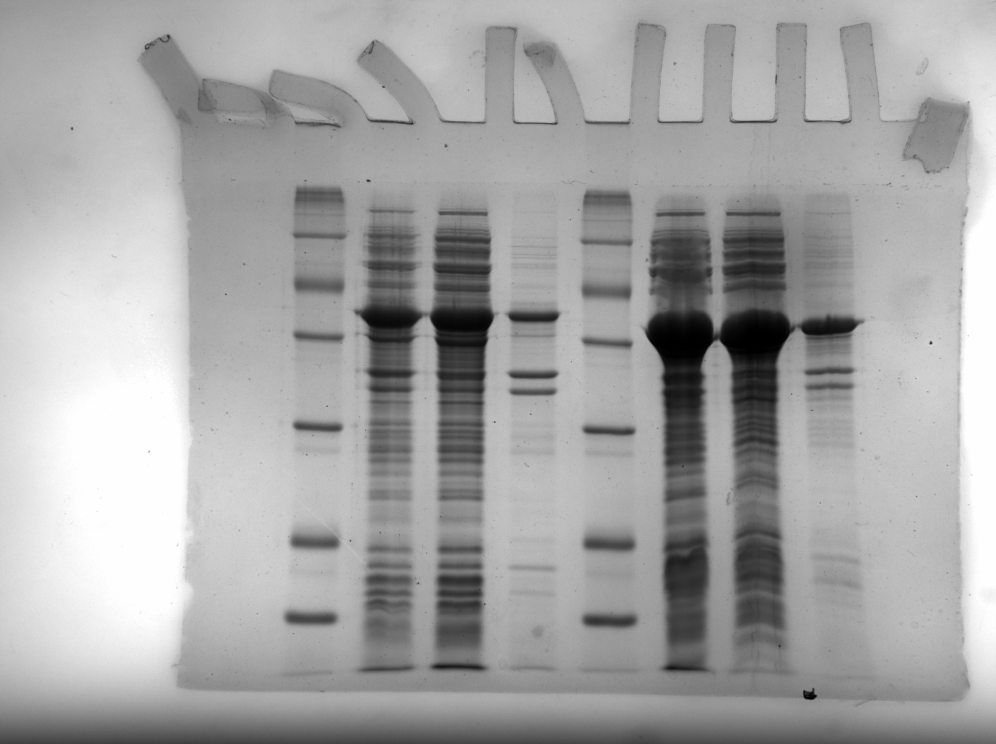
**

Fusion protein

**M 1 2 3**

**Fig B. Original image for protein bulk expression results.**

M, Marker; Lane 1, Whole bacteria after ultrasound treatment; Lane 2, Ultrasound induced supernatant; Lane 3, Ultrasonic precipitation. The rest are irrelevant samples

**
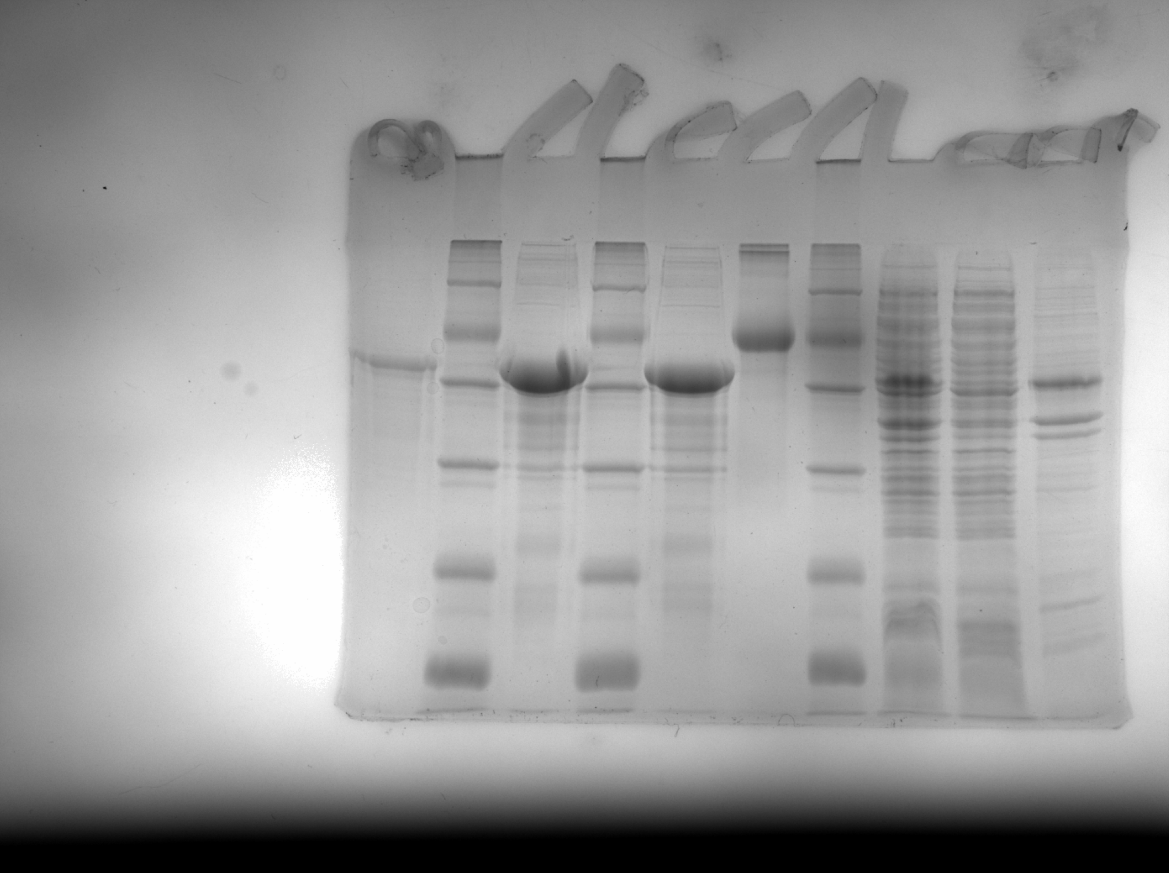
**

**1 M 2 M 3**

Fusion protein

**Fig C. Original image for protein purification results.**

M, Marker; Lane 1: Dilute purified protein threefold; Lane 2-3: purified protein stock solution. The rest are irrelevant samples





**Fig D. I-ELISA analysis of human serum samples (Integrate 126 negative serum and 283 cross-reactive samples).** (A) Dot plot of human serum samples. Fusion Protein Positive: OD_450_ values of positive sera tested with the fusion protein. Fusion Protein Negative: OD_450_ values of 126 negative sera and 283 cross-reactive samples tested with the fusion protein. LPS Positive: OD_450_ values of positive sera tested with LPS. LPS Negative: OD_450_ values of 126 negative sera and 283 cross-reactive samples tested with LPS. (B) ROC analysis of human sera.

**Table A. Evaluation of ELISA results of the recombinant proteins against positive, 126 negative sera and 283 cross-reactive samples**

| Antigen | AUC | Cut-off value | Sensitivity | Specificity | Positive | |  | Negative | | Accuracy  (%) | PPV  (%) | NPV |
| --- | --- | --- | --- | --- | --- | --- | --- | --- | --- | --- | --- | --- |
|  |  |  | (95%CI) | (95%CI) | TP | FN |  | TN | FP |  |  | (%) |
| Fusion Protein | 0.9930 (0.9892 to 0.9968) | >0.3950 | 0.9964 (0.9802 to 0.9999) | 0.9218 (0.8913 to 0.9459) | 278 | 1 |  | 377 | 32 | 95.20 | 89.68 | 99.74 |
| LPS | 0.9807 (0.9719 to 0.9895) | >0.3740 | 0.9391 (0.9042 to 0.9641) | 0.9438 (0.9168 to 0.9640) | 262 | 17 |  | 385 | 24 | 94.04 | 91.61 | 95.77 |

TP, true positives; TN, true negatives; FP, false positives; FN, false negatives; Accuracy, (TP+TN/TP+FN+TN+FP) ×100; PPV, positive predictive value (TP/TP+FP) ×100; NPV, negative predictive value (TN/TN+FN) ×100.
