# Supplementary material for: Multiepitope fusion protein-based ELISA for enhanced brucellosis serodiagnosis
Source: PLoS Negl Trop Dis. 2025 Dec 3;19(12):e0013804. doi: 10.1371/journal.pntd.0013804 (PMC12685213; doi:10.1371/journal.pntd.0013804)
Supplement: Attachment — Submitted filename: rebuttal letter.docx [file pntd.0013804.s005.docx]

Dear Editors,

Thank you for considering our manuscript. It is indeed a resubmission of the manuscript PNTD-D-25-00041. However, our manuscript has not been submitted for review. We have made the following revisions to the manuscript:

**1. Sample Size and Study Design**

In our original paper, there were only 164 brucellosis positive serum samples and 143 negative serum samples, including 27 non-brucellosis serum samples infected with other pathogens. In the revised manuscript, our sample size was expanded to 279 positive samples and 409 negative serum samples, including 283 non-Brucella patient serum samples infected with other pathogens. We have revised the Discussion section. In particular, it has clarified the limitations of our current research.

**2. Enhancement of Methodological Details**

We have expanded the Materials and Methods section to provide more detailed descriptions of the steps involved in the construction and evaluation of the fusion protein. This includes more detailed information on the selection of B-cell epitopes, the construction process, and the purification steps.

**Specific Changes:**

- Expanded the section on the selection of B-cell epitopes: "The identification of linear B-cell epitopes for Brucella was conducted using the Immune Epitope Database (IEDB) (<https://www.iedb.org/>) with the following basic parameters: Epitope type: Linear peptide; Assay type: B Cell; Outcome: Positive; Epitope Source: Brucella. All other parameters were maintained at their default settings. The retrieved B-cell epitopes were subsequently optimized for integration. In cases where amino acid sequences overlapped, the epitope was regenerated to ensure that the integrated epitope included the overlapping amino acid sequence."
- Added more details on the construction process: "The optimized integrated Brucella linear B-cell epitopes were tandemly linked, with adjacent epitopes connected by the linker sequence 'GGGS' to construct their corresponding amino acid sequences. Subsequently, the isoelectric point (PI) and molecular weight (MW) of the protein were predicted for the amino acid sequence of the tandemly linked fusion protein using ProtParam (<http://web.expasy.org/protparam/>) available on the ExPASy website. The physicochemical properties of the fusion proteins were thoroughly evaluated. Additionally, the three-dimensional molecular model of the fusion protein was predicted using I-TASSER (<http://zhanglab.ccmb.med.umich.edu/I-TASSER/>) to assess the spatial conformation of the fusion protein. The antigenicity of the fusion proteins was predicted and evaluated using VaxiJen software (<http://www.ddg-pharmfac.net/vaxijen/VaxiJen/VaxiJen.html>, threshold: 0.4, default)."

**3. Inclusion of Additional Data and Figures**

We have performed **BLAST analysis** for each epitope to confirm specificity to Brucella species (particularly B. melitensis and B. abortus). We also added supplementary information, including raw data for i-ELISA and original images for Figure 2, to provide more comprehensive support for our findings.

**Specific Changes:**

- Revised Table 1 (new column: Specificity) and added Supporting Information 4 (Blast results for each epitope)
- Added Supporting Information 2: Raw data for i-ELISA.
- Added Supporting Information 3: Original images for Figure 2.

**4. Statistical Analysis and Interpretation**

We have expanded the Statistical Methods section to provide a more detailed explanation of the statistical analyses performed and the interpretation of the results. We have also added a detailed explanation of the ROC curve analysis and the significance of the AUC values.

**Specific Changes:**

- Expanded the Statistical Methods section: "Dot plot and ROC curve analyses were conducted using GraphPad Prism version 6.05. Statistical analyses were performed using unpaired Student’s t-test, with a significance level set at P < 0.05. The ROC curve analysis was used to determine the sensitivity, specificity, area under the curve (AUC), and cutoff values for the fusion protein and LPS. The AUC values provide a measure of the overall performance of the diagnostic test, with higher values indicating better diagnostic accuracy."

We believe that these revisions have significantly improved the manuscript. We appreciate your consideration and hope that our revised manuscript is now suitable for publication in your journal.

Thank you once again for the opportunity to submit our work to your esteemed journal.

Sincerely,

Dehui Yin
