# Supplementary material for: Multiepitope fusion protein-based ELISA for enhanced brucellosis serodiagnosis
Source: PLoS Negl Trop Dis. 2025 Dec 3;19(12):e0013804. doi: 10.1371/journal.pntd.0013804 (PMC12685213; doi:10.1371/journal.pntd.0013804)
Supplement: Attachment — Submitted filename: Response to Reviewers.docx [file pntd.0013804.s006.docx]

**Dear Reviewers,**

We have studied all comments carefully and have made corrections. The comments and suggestions are constructive, and we implemented the recommended changes. Revised portions are marked in red in the paper. The main corrections in the paper and the responds to your comments are as flowing:

**Methods**

**Response to Reviewer 2**

Reviewer #2: The objectives are clearly stated and clearly addressed. Overall, the methods are thorough, but at times a bit verbose on accepted routine protocols (e.g. transformation). A few brief comments on the construction of the expression vectors would be helpful.
Sample size is appropriate and was clearly addressed from previous comments. Inclusion of positive controls, negative controls and samples from patients with other microbial infections was thorough.

**Response:** We have thoroughly revised the Methods section accordingly: We have streamlined the descriptions of routine protocols (e.g., transformation, protein expression, and purification) by removing excessive detail and referring to established methods and previous publications where appropriate. We have added a new subsection, **"2.5.1 Plasmid Construction"** which provides essential details on construction of the expression vectors.

**Response to Reviewer 3**

Reviewer #3: - Lines 128-129 - It is not clear how the presence of "varoius pathogens" was determined. The evaluation of cross-reactivity should be performed on samples that contains probable cross-reactive antigens, while here seems that the selection was made only on general diagnosis without testing sera for other pathogens antigens. The absence of a interferent agent don't allow to exclude cross-reactivity.

**Response:** We agree that using samples with confirmed non-brucellosis infections is essential. We have now revised the manuscript to clarify this issue. As suggested, we have added details in the Methods section (2.1) to specify that the sera from febrile patients were from cases with laboratory-confirmed infections of other pathogens (e.g., by microbial culture or PCR), and that the selection intentionally included pathogens known for potential cross-reactivity, such as *Escherichia coli* and *Klebsiella pneumoniae*.

Furthermore, we have also acknowledged the remaining limitation in the Discussion section (lines 375-382), noting that a future, more stringent validation could utilize panels with quantitatively confirmed antibody titers against specific cross-reactive agents.

- The sections 2.5.1 and 2.5.3 are in present tence, like a copy-paste from the experimental procedure: please modify to make in the same form of 2.5.2.

**Response:** We have revised these sections to ensure consistent tense usage throughout the Methods section, employing the past tense to describe the experimental procedures.

- Lines 249-250 - "who were not diagnosed": the description is incorrect and incomplete. The samples for cross-reactivity must be from individuals for which brucellosis was excluded and that risulted positive for other pathogens or negative at all. In the present form, it sound like the subjects had a possible infection with Brucella not confirmed in laboratory.

**Response:** We have now revised the manuscript to precisely define these samples. We have clarified in sections 2.1 and 2.6 that the sera used for cross-reactivity testing were explicitly from individuals whose brucellosis infection was excluded by a negative SAT result, and who were simultaneously positive for other laboratory-confirmed pathogens. This ensures that any reactivity observed in our iELISA can be confidently attributed to cross-reactivity with antibodies against non-*Brucella* pathogens.

**Results**

**Response to Reviewer 2**

Reviewer #2: Figure 1B is of questionable resolution to be informative, but all other figures and tables are clear and informative.

**Response:** At the time, I-TASSER provided prediction results in two formats: PDB and GIF. For convenience, we selected the latter. We have now re-predicted the protein's 3D structure and chosen to present it in PDB format. Please refer to the figure we have resubmitted for details.

**Response to Reviewer 3**

Reviewer #3: - Line 274 - This classifies the fusion protein as stable: why? The reader could not know how to interpret the index: what is the limit value to consider a molecule "stable"?

**Response:** We thank the reviewer for this suggestion. We have now clarified the instability index by adding the reference threshold (< 40 indicates stability) in the manuscript. The results output by the ExPASy server are as follows:

**Instability index:**

The instability index (II) is computed to be 33.46

This classifies the protein as stable.

- Sections 3.3 and 3.4 - These two paragraphs are very limited in information. Consider to add more details or to remove 3.3 and 3.4, that are currently useless. It looks like data were already present in existing authors' works reported in references.

**Response:** We thank the reviewer for this constructive criticism. We agree that these sections were overly concise. Instead of removing them, we have significantly expanded both sections 3.3 and 3.4 to provide essential and specific details of our current findings.

- Section 3.5 - How the reactivity against LPS by other pathogens was evaluated? As reported in Methods comments, it is necessary to confirm the presence of the antigen produced by other pathogens in the samples, otherwise a negative result could be attributed to the absence of the interfering substrate.

**Response:** We thank the reviewer for this exceptionally insightful comment, which gets to the very heart of a valid cross-reactivity assessment. We completely agree that without confirming the presence of antibodies against other pathogens in the test sera, the interpretation of the results would be ambiguous.

As now clarified in the Methods section (2.1), the serum samples from non-brucellosis febrile patients were not only SAT-negative but were also confirmed to have active infections from other pathogens via standard microbiological methods (culture or PCR). This critical piece of information, which we have now explicitly stated, guarantees that these sera contain the "interfering substrates" (antibodies against non-*Brucella* pathogens). Therefore, any reactivity observed in our iELISA is a true measure of cross-reactivity, and the lack thereof validly indicates low cross-reactivity potential.

**Conclusions**

**Response to Reviewer 2**

Reviewer #2: How does this change the outcome of serological detection of Brucella? How easily could this be implemented and what is the cost difference between LPS and purified recombinant multiepitope protein? How could this multiepitope sequence be further improved? What are the specific clinical implications? What is the significance of reduced false positives?
The conclusions are supported by the data and analysis. The limitations are sometimes too explicit (lack of Brucella information,

**Response:** We have thoroughly revised the **Discussion and** Conclusions **section** to directly address each of these points:

- We have added new paragraphs discussing the **clinical impact of reduced false positives**, the **practicality and cost-effectiveness of implementation,** the **specific clinical implications** of our fusion protein and **potential avenues for further improving the multiepitope sequence** in future research.
- As suggested, we have moderated the tone when discussing the study's **limitations**, reframing them more constructively as considerations for future research and clinical translation.

**Response to Reviewer 3**

Reviewer #3: - Lines 364-370 and 415 - There ae many discrepancies with data in other sections, that must be clarified. At present, these results do not include all samples collected and partially describe and support the study outcomes.

**Response:** We sincerely apologize. These data were samples collected from the original manuscript. Following the reviewers' comments, we increased the sample size and inadvertently omitted revisions to the discussion section. We have carefully verified the latest samples and made the necessary revisions.

- Lines 411-412 - "the fusion protein demonstrated comparable efficacy in recognizing positive samples when compared to LPS." Data on this are not presented in the work.

**Response:** We have therefore removed the claim of "comparable efficacy" and revised the sentence to more accurately reflect our findings.

**Editorial and Data Presentation Modifications?**

**Response to Reviewer2**

Reviewer #2: Line 39 Incomplete sentence.

**Response:** We have revised it.

Line 39, Line 58, Line 260, Line 265, Line 403, Table 1, conflicting statements of 23 epitope construct and 11 epitopes

**Response:** We sincerely thank the reviewer for this critical observation. The discrepancy arose from an insufficiently clear description of our epitope optimization process. To clarify:

- **23 epitopes** refer to the total number of individual, experimentally validated linear B-cell epitopes initially retrieved from the IEDB.
- **11 epitopes** refer to the final, non-redundant set of epitopes used to construct the fusion protein, which resulted from **merging the 23 initial epitopes where their amino acid sequences overlapped**.

We have now revised the manuscript **throughout** to consistently use this terminology and clearly explain the integration process. This ensures no ambiguity remains for the reader.

Line 279 The 3D structure of the protein is illustrated in Figure 1.

**Response:** We have revised it **(**Figure 1B)**.**

Line 428 change multiple hyphens to semicolon.

**Response:** We have revised it.
Line 440 typographical error "Aslo"

**Response:** We have corrected it.

Clarify what grayscale analysis means. What method was used to determine purity of the final protein?

**Response:** We thank the reviewer for this important question. We have now clarified the method in the manuscript. The purity of the purified fusion protein was determined through densitometric analysis using the professional Image Lab software from Bio-Rad. This analysis was performed on the Coomassie Brilliant Blue-stained SDS-PAGE gel image of the purified product (Figure 2C). We have updated both the Methods section (2.5.4) and the Results section (3.3) accordingly to provide this precise information.

Explanation of how the identity of the final protein was determined, possible reasons the observed MW of the protein is significantly different than the predicted size.

**Response:** We have added a discussion on the observed difference between the apparent molecular weight (~45 kDa) and the theoretical weight (35.1 kDa). We explain that this is a common phenomenon and provide several scientifically justified reasons, including the influence of **amino acid composition, physicochemical properties (e.g., low pI), and potential residual structure** on electrophoretic mobility in SDS-PAGE.

The manuscript could benefit from more explicit statements on the larger impact and significance of the research.

**Response: Based on your previous comments, we have added relevant content to the Discussion section.**

Was endotoxin (LPS contamination) measured in the final purified protein sample?

**Response:** The purified protein was subjected to endotoxin removal and subsequent quantification utilizing the Protein Endotoxin Removal Kit (C0268S, Beyotime, China) and the Endotoxin Detection Kit employing the Limulus Reagent Dynamic Turbidimetric Method (C0271S, Beyotime, China). We have added the details in 2.5.4 section and the result was added in 3.3 section.

**Response to Reviewer 3**

Reviewer #3: - Line 39 - Should the verb be "comprised"?

**Response:** In consideration of the suggestions provided by the other reviewer regarding the revision of this sentence, we have implemented the necessary modifications. The sentence should now conform to the required grammatical standards.

- Line 77 - Would it be possible to replace reference 2 with a more recent one? Perhaps using 3 also here.

**Response:** We have replaced Reference 2 with a more recent one.

- Line 83 - Cross-reaction is detected for many other pathogens, while here it seems that is limited to only these two. Please change (for example: cross-reactivity with other bacteria such as Yersinia ...).

**Response:** We have revised it.
- Line 84 - "contemporary research" has an unscientific soundness: please try to modify.

**Response:** We have revised it.
- Line 87 - "in contemporary research" is a repetition of previous sentence: please modify.

**Response:** We have revised it.
- Lines 89-91 - Make the sentence more linear and clear.

**Response: We have revised it.**
- Line 91 - "In this methodology,": remove and add and adverb in the sentence (for example: ...proteins is therefore of...).

**Response:** We have revised it.
- Line 94 - The work in reference 12 did not conclude the superior performance of LPS.

**Response:** Based on your comment, we have updated the reference.
- Lines 99-101 - A reference is needed.

**Response:** We have added the relevant references.
- Line 129 - "which collected": a verb is missing.

**Response:** We appreciate your comment. In response to feedback from other reviewers, we have revised this section accordingly.

- Line 265 - "Refer to" in brackets is not necessary.

**Response:** We have deleted it.
- Lines 266-268 - It would be better to change "are particularly" in something like "was found to be conserved among most relevant species..."

**Response:** We have revised it.
- Line 304 - Is it "Cause of human"?

**Response:** We have revised it.
- Lines 423-424 - It is impossible to deduce species from sera with the available information: it would be better to explain that the specie infecting serum donors was not determine.

**Response:** We have revised it.
- Lines 434-436 - Please put in evidence what is the diffrence with previous works of the authors.

**Response:** Thank you for your comment. We fully agree that explicitly stating the advance over our previous work strengthens the manuscript. In fact, the key difference from our previous work, which relied on *in silico* prediction tools, is the source of the epitopes. Here, we constructed the fusion protein exclusively from experimentally validated linear B-cell epitopes sourced directly from the IEDB, in contrast to the bioinformatically predicted epitopes used in our earlier studies. This strategy enhances the reliability of epitope selection by building upon a foundation of prior experimental evidence. As suggested, we have now **added a clear statement** to highlight the key difference.

**Summary and General Comments**

**Response to Reviewer 1**

Reviewer #1: Abstract: the sentence ‘lower specificity, but less cross-reactivity’ seems a paradox
**Response:** We sincerely thank the reviewer for this astute observation. The apparent paradox stems from an imprecise description of our negative control cohort in the original manuscript, which we have now corrected. The 'negative' sera were not from healthy individuals, but from febrile patients suspected of brucellosis but confirmed to be SAT-negative. This cohort is therefore enriched with samples that pose a high risk for cross-reactivity. The fusion protein does not have "lower specificity"; rather, it demonstrates superior and more clinically meaningful specificity by effectively distinguishing brucellosis from other febrile illnesses, which is the central diagnostic challenge. We have revised the Abstract and Methods sections to reflect this clearly.

Author’s summary:

Line 64-65: the numbers in these sentences contradict the results in the abstract
**Response:** We sincerely thank the reviewer for this critical observation. We apologize for this inconsistency, which was an oversight during the manuscript preparation. We have rechecked the original data and thoroughly corrected this portion of the data. We have now corrected this error throughout the manuscript. The changes are highlighted in the revised manuscript. Fusion protein: **9/283** cross-reactive samples. LPS: **41/283** cross-reactive samples.

Line 83: Yersinia enterocolitica O9 and Escherichia coli O157:H7: where these included in controls?
**Response:** We thank the reviewer for raising this important point. The two samples, which are essential for key validation, should have been incorporated into the control group. Unfortunately, these samples were not collected in the clinical setting. This limitation has been acknowledged and discussed in our manuscript.

Line 125: had positive controls also positive blood cultures? If so, which Brucella species were found? If not, how certain are the authors that these were true Brucella samples? Was there seroconversion? A single positive serology test does not provide an accurate diagnosis of brucellosis.
**Response:** We thank the reviewer for these critical questions regarding the certainty of our positive brucellosis samples. Firstly, our samples were not cultured for verification because Brucella culture necessitates a Biosafety Level 3 (BSL-3) laboratory, which is not available to us. Secondly, in China, brucellosis is designated as a Category B notifiable infectious disease, and all confirmations are performed at disease control centers using serological methods: initial screening is conducted via the RBPT, followed by confirmation with the SAT. In any case, the samples we collected do indeed have significant limitations. We have acknowledged and clarified this limitation in the discussion section of our manuscript.

Table 2: the abstract says ‘9’ cross reactivity in the fusion protein group, but there are only ‘8’ FPs; there are ‘39’ that showed cross reactivity in the LPS group, but only ‘2’ FP in the table
**Response:** We thank the reviewer for this close scrutiny of the data. The apparent discrepancy arises because the numbers come from two distinct and independent serum panels used to evaluate different aspects of assay performance. We apologize for any confusion our original description may have caused. Please allow us to clarify:

The 'FP' in Table 2 (2 for LPS; 8 for Fusion Protein) comes from the negative control group (n=126), which consisted of sera from - febrile patients in whom brucellosis was ruled out. These 'FP' values were used to calculate the Specificity of the assay. The 'cross-reactivity' numbers in the Abstract come from a separate, larger panel of sera (n=283) from patients with laboratory-confirmed active infections by other, non-Brucella pathogens. This panel was used specifically to test for cross-reactivity.

In summary, specificity was assessed against a general negative control group. Cross-reactivity was specifically assessed against a challenging panel of sera from patients with other known infections.

It is confusing that in the methods section, line 126 describes healthy individuals as controls, and on line 128, samples from febrile patients with pathogens are used to check for cross-reactivity. It should be noted that the latter group is, in the real world, a much more realistic and effective control group than the first group. One can question why the Table 2 and ROC curve were not created with samples from febrile patients with pathogens as the control.
**Response:** During the initial phase of our study on brucellosis diagnosis, we lacked samples necessary for assessing cross-reactivity. However, following reviewer recommendations during the submission process, we sought to incorporate these results. In consultation with experts in epidemiology and statistics, we clarified that specificity and cross-reactivity represent distinct concepts: specificity pertains to analytical specificity within the methodological framework, whereas cross-reactivity relates to clinical diagnostic specificity. For ROC curve analysis, the control group may comprise sera from individuals without brucellosis. Nonetheless, we recognize that this approach may lead to an overestimation of assay performance in complex clinical settings. To address this important concern and to provide a more rigorous and clinically relevant evaluation, we conducted an additional ROC analysis using sera from febrile patients infected with other pathogens (n = 283) as the control group. The results of this analysis have been incorporated as Supplementary Table S1 and Supplementary Figure S4 and are now cited in the main text (Results section 3.4).

In the results section, tables, and figures, it is not clearly stated which of the two control groups was used, which is critical for correct interpretation. Also, in the abstract, it is not possible to know that there are actually two different control groups, and the results section mixes up both.
**Response:** We sincerely thank the reviewer for this insightful and crucial comment. We agree that the distinction between the two control groups is fundamental to the interpretation of our diagnostic accuracy and cross-reactivity results. We apologize for the lack of clarity in the original manuscript. We have now thoroughly revised the text to explicitly define and differentiate the two control groups throughout the manuscript, including the Abstract, Methods, Results, and Figure Legends.

Discussion:
Explain the role of the O-antigen in the rough vs smooth Brucella species
**Response:** According to your suggestion, we have added the relevant content in the first paragraph of the discussion.

The discussion is lengthy, and many paragraphs discuss issues related to the specific antigens, which are less relevant to the discussion. The core statistical findings, along with their strengths, limitations, and implications for clinical practice, are not discussed.
**Response:** According to your comments and those of other reviewers, we have made substantial revisions to the discussion section.

**Conclusion:**
Limitations raised here are part of the discussion, not the conclusion. The findings should be summarized as well as their importance.

**Response:** In accordance with this comment, we have revised and created a New, Concise Conclusion.

**Response to Reviewer 2**

Reviewer #2: The strongest section is the discussion section, and the manuscript could benefit from expanded analysis of other sections.

**Response:** According to your comments and those of other reviewers, we have made substantial revisions to the discussion section.

**Response to Reviewer 3**

Reviewer #3: The work was well written and understandable. However, crucial data are missing, in particular regarding testing for cross-reactivity. The authors have other works with similar topics, but the data therein presented and of interest for the present paper must be included: a reader must have all data needed in the single work.

**Response:** We sincerely apologize for any confusion regarding the availability of the cross-reactivity data. We would like to clarify that the detailed, raw data for the cross-reactivity testing, which includes the OD450 values for all 283 samples from non-brucellosis patients tested against both our fusion protein and the LPS control, is fully provided in Supporting Information 2 (File Name: Supporting information 2. Raw data for I-ELISA.xlsx).

Once again, thank you very much.

Best wishes,

Dehui Yin
