# Supplementary material for: Multiepitope fusion protein-based ELISA for enhanced brucellosis serodiagnosis
Source: PLoS Negl Trop Dis. 2025 Dec 3;19(12):e0013804. doi: 10.1371/journal.pntd.0013804 (PMC12685213; doi:10.1371/journal.pntd.0013804)
Supplement: Attachment — Submitted filename: Response_to_Reviewers_auresp_2.docx [file pntd.0013804.s007.docx]

**Dear Reviewer 1**,

We have studied the comments carefully and have made corrections. We thank you for this precise and insightful comment, which helps us to articulate our findings with greater accuracy. You are absolutely correct. The key advantage of our fusion protein—a significant reduction in false positives—is specifically demonstrated when differentiating brucellosis from other confirmed bacterial infections (particularly Gram-negative ones), which are the primary source of cross-reactivity with LPS. The samples we use to validate cross-reactivity do not represent the real world. The main corrections in the paper and the responds to your comments are as flowing:

Q1: Line 369: to be clarified that fewer false positives were in the comparison with confirmed bacterial infections, which will have more Gram-negative bacteria that cross-react with LPS. In the ‘febrile’ control group, which might better reflect the real world, there were no fewer false positives.

**Response:** We have revised the relevant sentence in the Discussion to reflect this crucial distinction. (Lines 370-374).

Q2: Line 433: same comment as above. Real-world febrile patients are not synonymous with confirmed bacterial positive samples. In the ROC curve, representing real-world ‘febrile’ patients, LPS outperformed the fusion protein

**Response:** We have revised the manuscript in its entirety based on your comments. (line 361, lines 378-387)

Q3: Line 490: cross-reactivity with bacteremic infections (cfr above)

**Response:** We have revised it based on your comments. Meanwhile, we have also made similar revisions to our manuscript, such as line 39, line 46, line 51, line 69 and line 446.

Once again, thank you very much.

Best wishes,

Dehui Yin
